# Obstructed Hemi-Vagina with Ipsilateral Renal Agenesis Syndrome in Adulthood: A Diagnostic Challenge

**DOI:** 10.3390/diagnostics13213377

**Published:** 2023-11-03

**Authors:** Alexandros Loukas Grammatis, Femi Ajibade, Dinuke Warakaulle, Tunde Dada

**Affiliations:** 1 Department of Obstetrics and Gynaecology, Oxford University Hospitals NHS Trust, Oxford OX3 9DU, UK; 2 Department of Obstetrics and Gynaecology, Cumberland Infirmary Hospital, Carlisle CA2 7HY, UK; femiajibademd@doctors.org.uk; 3 Department of Radiology, Buckinghamshire Healthcare NHS Trust, Aylesbury HP21 8AL, UK; dinuke.warakaulle@nhs.net; 4 Department of Obstetrics and Gynaecology, Buckinghamshire Healthcare NHS Trust, Aylesbury HP21 8AL, UK; tunde.dada@nhs.net

**Keywords:** OHVIRA syndrome, differential diagnosis, tubo-ovarian abscess, congenital syndrome, pelvic inflammatory disease, MRI

## Abstract

A patient in her early 20s presented with constant and progressive lower abdominal and back pain, mainly on the right side of the abdomen, purulent vaginal discharge and pyrexia. A radiological assessment revealed a possible tubo-ovarian abscess and the incidental diagnosis of ipsilateral renal agenesis. The patient was treated for pelvic inflammatory disease (PID); however, after antibiotic administration and since the symptoms did not resolve, an abdominal MRI was requested, which revealed uterus didelphys with two cervices, an obstructed haemivagina and evidence of haematocolpos. The diagnosis of Obstructed Hemi-Vagina with Ipsilateral Renal Agenesis (OHVIRA) syndrome was confirmed, and the patient underwent the excision of the vaginal septum, the drainage of the haematopyocolpos and the laparoscopic drainage of the tubo-ovarian abscess. She achieved a good recovery.

**Figure 1:**
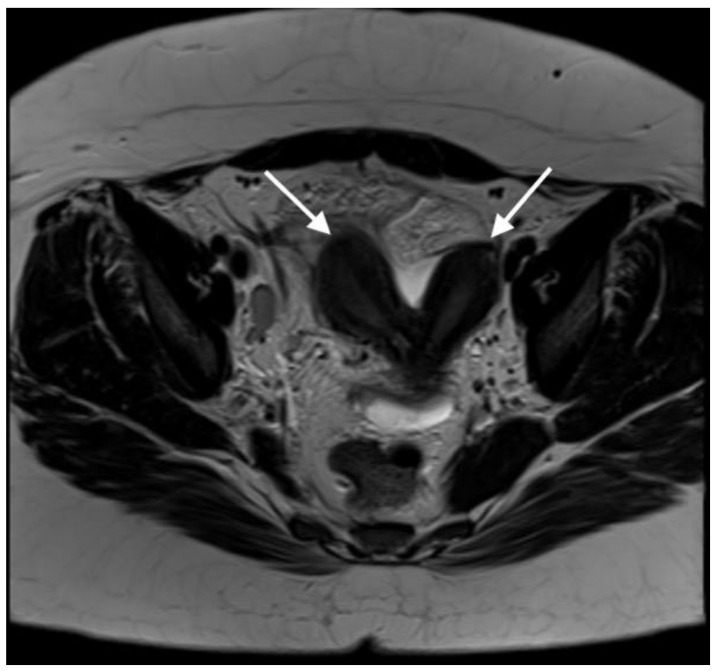
**Axial T2-weighted MR image demonstrates uterus didelphys (two uterine cavities indicated by white arrows).** A 21-year-old woman presented with lower abdominal and back pain, mainly located on the right side. The pain was constant and progressively worsened over a six-day period. She also reported episodes of vomiting over the last two days, constipation and a loss of appetite. Additionally, she had a previous assessment in A + E five days prior to this admission, where she was diagnosed with a chest infection and was treated with Co-amoxiclav. Her previous medical history and family history were unremarkable, she had never had any previous surgeries, had never conceived and was not allergic to any medication. Initially, she was assessed by members of the A + E team and was referred to the surgical team to exclude appendicitis or mesenteric adenitis. Upon examination, her abdomen was soft with voluntary guarding on the right iliac fossa. She was pyrexial (T: 38.1 °C), tachycardic (HR: 129 bpm), tachypnoeic (RR: 19), with normal blood pressure (BP: 119/80 mmHg) and oxygen saturation levels (SO_2_: 98%). Biochemical and haematological investigations revealed elevated CRP (>320 mg/L), white cell count (WCC: 16 × 10^9^/L) and platelets levels (405 × 10^9^/L). The liver and kidney functions were normal. Venous blood gas indicated a pH of 7.35 and lactate levels of 1.3 mmol/L. The urine sample indicated the presence of leucocytes, protein and blood. The urine pregnancy test was negative. The patient underwent a CT of her abdomen/pelvis after the initial surgical review. It revealed a large complex pelvic collection, measuring 7 × 6 × 10 cm, arising from the right adnexa, with the inflammatory process extending into the uterus. The CT also suggested incidental right renal agenesis, which the patient was not aware of. The findings were suggestive of a tubo-ovarian abscess (TOA), and the patient was started on intravenous ceftriaxone and metronidazole, as per the unit’s protocol. Due to the CT scan findings, the patient was then referred to the gynaecology team. Her gynaecological history indicated a menarche at 10 years of age. Her periods were regular, but she reported significant dysmenorrhea and heavy menstrual bleeding. Clinical examination including a speculum exam revealed that the cervix was difficult to visualise, since it was deviated to the left. A purulent foul-smelling discharge was also noted as well as a fullness on the anterior fornix, but no signs of significant tenderness were present at the time of the examination. After the gynaecological assessment, a pelvic ultrasound was requested to further characterize the pelvic mass. TVUSS was performed approximately 24 h after the CT, while the patient remained febrile. The sonographic assessment revealed the collection, previously attributed to the TOA, but the additional finding of uterus didelphys with two cervices was also noted. The combination of renal agenesis and uterine abnormality raised the possibility of a Müllerian abnormality; thus, an abdomen/pelvis MRI with contrast was requested in order to confirm the diagnosis. The MRI report confirmed that there was a double uterus and cervix and further added that there was a longitudinal vaginal septum (U3bC2V2 based on the ESHRE/ESGE classification [1]).

**Figure 2:**
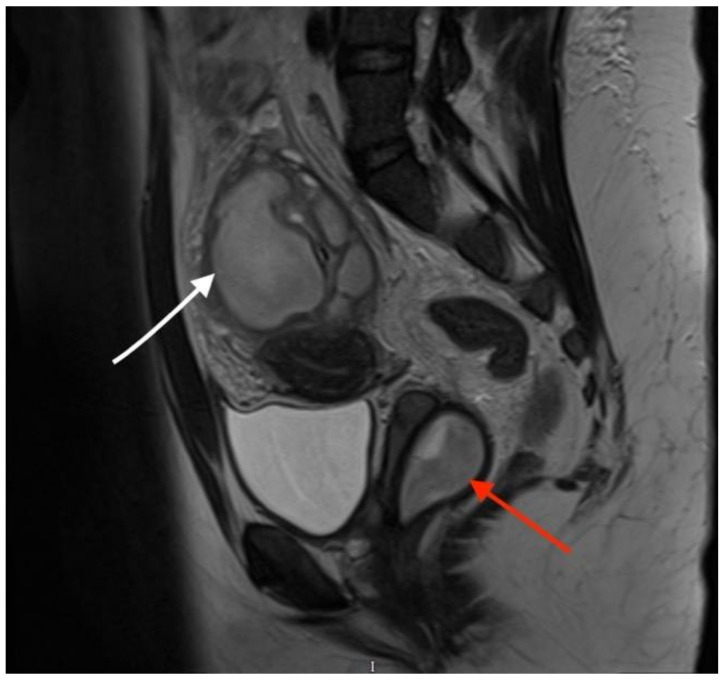
**Sagittal T2-weighted MR image showing a vaginal septum with a haematopyocolpos of the obstructed hemivagina and a tubo-ovarian abscess (red arrow indicates the obstructed haematopyocolpos, and white arrow shows the tubo-ovarian abscess)**. The presence of gas and fluid within the lumen was indicative of a concurrent infection. The patient was initially treated with intravenous antibiotics after the provisional diagnosis of PID. After three days of treatment, she continued to have episodes of pyrexia, the CRP level remained high (242 mg/L) and there were persistently high WCC (16.5 × 10^9^/L) and platelet quantities (500 × 10^9^/L). On the third day after admission, the diagnosis of OHVIRA syndrome was finalised, and the decision was taken to take the patient for surgical intervention to drain the haematocolpos and perform a laparoscopic exploration, to which the patient consented. The next day, the patient underwent an examination under anaesthesia, vaginal septectomy and drainage of the haematopyocolpos. Intraoperative hysteroscopy confirmed the presence of a uterus didelphys bicollis. The cervix of the affected cavity was dilated to encourage continuous drainage. Subsequently, during the laparoscopy, significant pelvic adhesions were noted, involving the bowel being adherent to the uterus, and a large right-sided tubo-ovarian abscess. Laparoscopic adhesiolysis was performed, as well as the drainage of the abscess. The pelvic drain was left in situ at the end of the procedure. She remained an inpatient for four days after surgery. The drain was removed three days after the operation. She was continued on intravenous antibiotics for a further three days before they were converted to oral medication. She no longer had a post-operative spiking temperature, and her pulse normalised two days after the operation. Her CRP dropped to 51 mg/L and WCC to 9.5 × 10^9^/L on the day of discharge. She was followed up in the Gynaecology Clinic with repeat imaging that confirmed the resolution of the tubo-ovarian abscess. She was carefully debriefed about the implications of having uterus didelphys and a single kidney in pregnancy and was further signposted to written information provided by the British Society of Paediatric and Adolescent Gynaecology.

**Figure 3:**
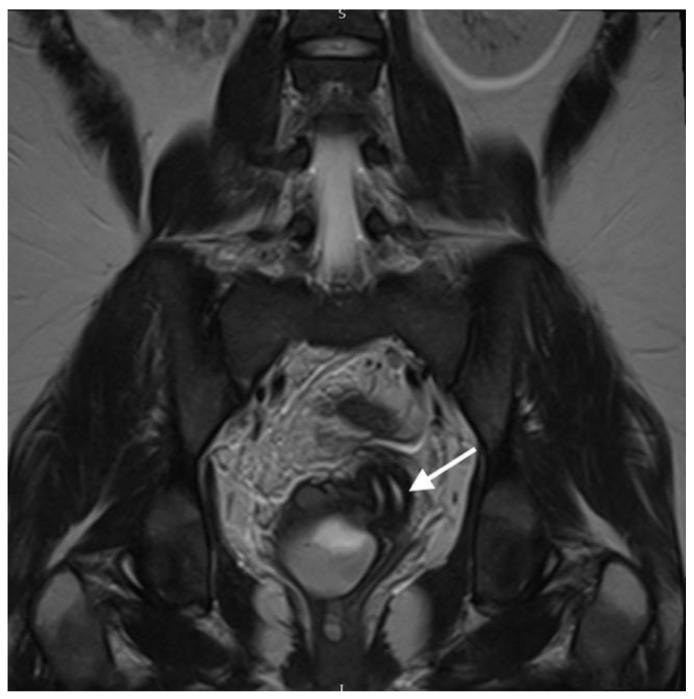
**Coronal T2-weighted MR image shows 2 cervical canals (White arrow indicates the two cervical canals. The right-sided cervix leads to the obstructed haematopyocolpos)**. OHVIRA syndrome, also known as Herlyn–Werner–Wunderlich syndrome, is a rare uterine and renal congenital abnormality which is characterized by an obstructed hemivagina and ipsilateral renal agenesis [2]. The exact prevalence of the syndrome has not been yet estimated, but it has been reported that it accounts for 0.16–10% of Mullerian abnormalities, the incidence of which is 2–3% of the population [3]. Assessment via pelvic ultrasound in combination with MRI helps to establish an accurate clinical diagnosis [4]. In the majority of cases, pubertal women are more likely to be identified with the syndrome, since the onset of menstrual periods might lead to hematometra and/or delayed menarche. However, in cases of partial obstruction, the diagnosis can be elusive, often taking years to be established, leading, at times, to women presenting in adulthood with symptoms resembling pelvic infection and endometriosis [2]. Septectomy remains the treatment of choice and should be performed in all affected patients. Knowledge of this rare condition and maintaining high levels of suspicion is critical in these cases [5,6].

The pathogenesis of OHVIRA syndrome derives from the anomalous development of the paramesonephric (Müllerian) and mesonephric (Wolffian) ducts during the early stages of embryological development [6]. The uterus, cervix and both fallopian tubes derive from the Müllerian ducts [7]. In the eighth week of pregnancy, the paired Müllerian ducts that derive from invaginations of the coelomic epithelium vertically fuse. The fused cranial ends give rise to the left and right parts of what eventually will become the uterus. The unfused cranial ends of the ducts will form the fallopian tubes. While this process is complete by the end of the first trimester, a thick midline septum is present along the uterus, cervix and vagina that will normally reabsorb by 20 weeks of pregnancy [4]. A failed or incomplete fusion will lead to uterus didelphys or a bicorporeal uterus, whereas the non-reabsorption of the septum will lead to a septate uterus. The origin of the vagina is less clear. According to the classic theory [8], the upper part of the vagina develops from the caudal end of the fused Müllerian ducts, and the lower part develops from the sinovaginal bulbs derived from the urogenital sinus. However, this theory is insufficient to explain the development of intricate syndromes like OHVIRA, characterised by a combination of uterine, vaginal and renal anomalies. Acien et al. [9] have proposed that the vaginal derives solely from the distal Wolffian ducts, with its only its lining stemming from the Mullerian tubercle. Concurrently, the failure of the metanephric diverticulum to arise from the Wolffian duct leads to the agenesis of the ureteric bud, and subsequently, the agenesis of the ipsilateral ureter and kidney. Thus, the anomalous development of one of the Wolffian ducts will lead both to an obstructed hemivagina and renal agenesis, making OHVIRA syndrome a mesonephric-duct induced Müllerian anomaly. This theory has been supported by experimental studies on female rats [10].

This complex embryological origin makes OHVIRA syndrome a complicated, heterogeneous condition that can be challenging to diagnose. The common triad of congenital variations (uterus didelphys, an obstructed hemivagina and renal agenesis on the same side of the vaginal abnormality) is not always present, as reports have indicated that there can also be a septate uterus in up to 22% of cases [11]. The renal anomalies can also include an ectopic ureter or a dysplastic kidney [12,13]. Variants of the syndrome have also been reported, where there is a fenestration in the vaginal septum that results in a partial obstruction [6], or cases when the septum is very thin, and menstrual contents cause it to perforate leading to communication between the two vaginas [14]. Patients with this type of vaginal variation, such as in our case report, will have normal menses through the patent hemivagina and will typically have a delayed diagnosis much later during their reproductive years. While the classic clinical presentation is that of a prepubertal girl around menarche presenting with pelvic pain, and a vaginal bulge is present upon examination, indicating haematocolpos, cases that present in adulthood can be misdiagnosed with more common conditions such as pelvic infection, tubo-ovarian abscess or endometriosis. In adulthood, patients will usually present with progressive dysmenorrhea and lower abdominal pain and will also possibly report urinary retention and offensive vaginal discharge [15]. On an acute basis, they might present with symptoms of an acute pelvic infection, which are secondary to pyocolpos, leading to a pyometra and tubo-ovarian abscess. These cases can closely resemble the symptoms of PID, and clinicians will need a high index of suspicion as the patients will not respond to antibiotics. Furthermore, endometriosis, in the form of ovarian or deep infiltrating endometriosis, is found in 17–36% of these patients [16,17], with the most likely pathogenesis being the inability of the obstructed hemivagina to distend due to the thick septum resulting in retrograde menstruation [18]. Other studies indicate that the age of the diagnosis of pelvic endometriosis usually comes much earlier than the diagnosis of OHVIRA syndrome [19], and clinicians should be aware that patients with Mullerian anomalies can develop severe endometriosis, particularly in cases with an obstructed outflow tract [20]. Thus, in adolescent girls diagnosed with pelvic endometriosis, due to symptoms of dysmenorrhea with an onset close to menarche, an obstructive genital abnormality should be considered as a potential diagnosis [2]. First and foremost, ultrasound can assist in diagnosis. Some authors report that a transvaginal ultrasound is sufficient to diagnose the syndrome [21,22]; although, particularly in adolescents when only an abdominal ultrasound can be performed, it is highly dependent on the sonographer’s skill and expertise. An abdomen/pelvis CT scan is usually readily available in most units; however, it cannot depict the vagina as accurately as an MRI, and it exposes patients to radiation [4]. Most studies agree that an MRI is the gold-standard investigative tool to diagnose the syndrome and to accurately plan a surgical intervention [23].

OHVIRA syndrome is a rare Mullerian syndrome, with more than 700 cases reported in the literature, mostly from tertiary units; however, this small number could be due to underdiagnosis related to the poor awareness of this condition [24]. Clinical examinations are often not reliable, leading to incorrect diagnoses upon presentation; therefore, clinicians should maintain a high index of suspicion to diagnose such cases in a timely manner. It is worth noting that, in this case, the CT scan did not accurately diagnose the uterine anomaly but indicated the incidental unilateral renal agenesis. Studies have shown that over 30% of female patients with a unilateral renal agenesis or multicystic dysplastic kidney have an associated uterine anomaly, so such a finding should always trigger further imaging of the uterus either via ultrasound or MRI [24]. Screening young women, before menarche if possible, with such renal abnormalities for Mullerian anomalies would potentially prevent, not only acute presentations such as the one we describe in this case, but also the long-term complications associated with untreated obstructive malformations. Clear guidelines are currently scarce to guide this type of screening, but they are needed to ensure adequate communication between sonographers, paediatricians, and gynaecologists to diagnose this syndrome early [24]. They would also help to promote the age-specific treatment of the vaginal obstruction [25]. Studies on the long-term reproductive outcomes of patients with OHVIRA syndrome indicate that a delayed diagnosis is associated with high rates of PID, pelvic adhesions and endometriosis, although thankfully, not subfertility [11,26]. Pregnancies are, in up to 80% of cases, on the contralateral side of the obstructed hemivagina, but they have also been reported on the same side [11]. High rates of preeclampsia (14%), preterm delivery (36%), breech presentation (38%) and a high caesarean section rate (67%) are common obstetric complications found in patients with OHVIRA syndrome, and the patients should be appropriately counselled [11,26,27]. The treatment as described in this case involves a simple correction of the vaginal obstruction via vaginal septectomy. This will relieve the menstrual flow and spontaneously drain the haematocolpos, haematometra and haematosalpinx. Some authors advocate that the septectomy should be followed by the complete resection of the vaginal septum and vaginal remodelling depending on the thickness of the septum either as a single- or two-stage procedure [28]. In any case, a simple septectomy is highly effective to relieve dysmenorrhea and can also achieve the resolution of endometriosis if present [29]. The early diagnosis of the condition can prevent aggressive interventions with long-term reproductive implications, such as salpingectomy and hemihysterectomy, that are used to treat the delayed complications of the obstruction, such as a tubo-ovarian abscess and pyometra [11,28].

This case clearly demonstrates the challenges clinicians can have in diagnosing this condition in adult patients and how crucial cooperation and communication between clinicians from different specialties is. An increased awareness of the symptomatology of this syndrome can lead to a timely diagnosis and prevent years of dysmenorrhea and chronic pelvic pain, as well as complex surgical interventions with significant morbidity for the patients. Clear guidelines could ensure the prompt diagnosis and treatment of OHIVRA syndrome in the majority of cases, starting from the postnatal screening of uterine anomalies for babies that were diagnosed with renal agenesis and leading to age-specific treatment based on the specific anomaly [24,25,30].

## Data Availability

All data generated or analysed during this study are included in the article.

## Abbreviations

OHVIRA	Obstructed Hemi-Vagina with Ipsilateral Renal Agenesis
PID	Pelvic inflammatory disease
TOA	Tubo-ovarian abscess
CT	Computed tomography
MRI	Magnetic resonance imaging
CRP	C-reactive protein
WCC	White cell count
T	Temperature
RR	Respiratory rate
HR	Heart rate
mg/L	milligrams per litre
mmol/L	millimole per litre
SO_2_	saturation of oxygen
